# Effects of Mirror Therapy Combined with EMG-Triggered Functional Electrical Stimulation to Improve on Standing Balance and Gait Ability in Patient with Chronic Stroke

**DOI:** 10.3390/ijerph18073721

**Published:** 2021-04-02

**Authors:** Dong-Hoon Kim, Sang-Hun Jang

**Affiliations:** 1 Department of Physical Therapy, Gimcheon University, 214, Daehak-ro, Gimcheon 39528, Korea; roopi00yo@naver.com; 2 Department of Physical Therapy, College of Health and Life Science, Korea National University of Transportation, 61, Daehak-ro, Jeungpyeong-gun, Chungbuk 27909, Korea

**Keywords:** stroke, EMG-FES, mirror therapy, balance, gait

## Abstract

This study was performed to evaluate the effects of EMG-triggered functional electrical stimulation on balance and gait ability on patient with Chronic Stroke. A total of 60 chronic stroke patients were divided into mirror treatment and functional electrical (MT-EF) Group, MT group, CON group. Each group performed 60 min a day five times a week for eight weeks. MT-FE group was performed 30 min five times a week for eight weeks in mirror therapy process with EMG-FES. MT group performed 30 min five times a week for eight weeks in mirror therapy process. CON group was performed 30 min five times a week for eight weeks in conservative treatment. To measure the balance ability, Biorescue (COP, LOS), Berg balance scale (BBS) and FRT, and the gait ability test was performed by 10 m walk test. MT-FE group revealed significant differences in COP, LOS, BBS, FRT and 10 m walk test as compared to the MT and CON groups (*p* < 0.05). Our results showed that MT-FE was more effective on COP, LOS, BBS, FRT and 10 m walk test in patients with chronic stroke. Our results also showed that MT-EF group was more effective on balance and gait ability in patients with chronic stroke. We suggest that this study can be used for intervention data for recovering balance and gait ability in chronic stroke patients.

## 1. Introduction

Stroke is a chronic disease due to ischemia and bleeding in brain tissues. It can cause movement disorder, visual defects, sensory impairment, speech loss, and sequelae, such as intellectual disability, thus, degrading the quality of life of patients and their families. With the onset of stroke, brain cells start to die due to insufficient oxygen supply [1]. Cell death will lead to the loss of ability for some areas of the brain that control cognitive and muscle functions [2].

Balance is the ability to maintain the center of gravity within the base of surface. It can continuously maintain the body posture in response to changes in the environment when the body’s weight is moved [3]. Stroke patients have difficulties in controlling this balance as postural fluctuations are increased in a standing position. In addition, as the weight load on the lateral side increases, it is difficult to distribute the same weight on both sides, thereby reducing their ability to move a posture [4]. In addition, as their postural difficulty (sitting, standing, walking, climbing stairs) increases, it is difficult for stroke patients to perform tasks [5]. Rehabilitation for such stroke patients can improve their balance and make them achieve functional movements [6]. Since balance consists of various elements, it is difficult to improve the balance ability of stroke patients [7].

Methods used to improve balance and gait of stroke patients include training using functional electrical stimulation [8], dual task exercise [9], therapeutic approach through collaborative action of Brunstrom [10], virtual environment rehabilitation [11], mirror therapy [12], and so on. Among them, mirror therapy is one of treatments for patients with brain lesions based on the theory of neuroplasticity in the brain. This method focuses on the movement of limbs on the non-paralytic side to promote recovery of motor function [13].

In mirror therapy, the motion of the non-injured limb reflected in the mirror is projected as the motion of the paralyzed limb. This treatment involves the theory that images of elevated movements, given by visual information that is superimposed on the paralyzed limb, stimulate the brain [14]. Functional brain imaging studies have revealed that the reflection of one hand in the mirror can excite the primary motor area of the brain, which is connected to the other hand, even in normal people [15].

Functional Electrical Stimulation (FES) is a method that is widely applied to recover the function of stroke patients. Using this this method, an electrical stimulation is applied to the paralyzed or weakened muscle to induce muscle contraction. Through this process, it is possible to prevent muscle atrophy, maintain muscle tolerance, strengthen muscle strength, and retrain functional movements [16,17]. Kroon and IJzerman [18] have stated that, unlike simple repetitive stimulation, such as conventional electric stimulation, improvements in symptoms require an active will and motivation of the patient to move the paralyzed side by himself/herself. EMG (electromyography)-linked electrical stimulation is a method of inducing muscle contraction by delivering electrical stimulation to peripheral nerves when an electrical signal, generated by a patient’s voluntary movement effort, exceeds a threshold value set in the EMG. The reasons why EMG-Triggered electrical stimulation can exhibit a better effect than simple electrical stimulation are described as follows. First, repetitive passive exercise by simple electric stimulation does not involve concentration or eccentric motor nerve mobilization by cognitive function. However, during EMG-triggered electrical stimulation, there is a continuous increase in concentration to move muscles used. Second, a weak activation of the motor function occurs so that patients can move target muscles willfully. Third, this method can be applied to the theory of sensorimotor integration that is simultaneously or sequentially integrated in the cerebrum. Examples include feedback of proprioceptive sensation by movement of target muscle, transmission of somatosensory electrical stimulation, and so on [19].

Studies on mirror therapy and functional electrical stimulation have been reported. Galeazzi et al. [20] have reported that the balance ability is improved by performing mirror therapy with task training through a full-body mirror in patients with chronic stroke. Lee et al. [21] have enrolled an electric stimulation with mirror therapy training group, and a sham electric stimulation, with mirror therapy training group of patients with chronic stroke. In patients with stroke, Kojima et al. [22] conducted a study to determine whether immediate EMG-Triggered functional electric stimulation training and delayed EMG-Triggered functional electric stimulation can significantly improve wrist flexor stiffness. Ji and Kim [23] conducted a study, which confirmed that gait ability can be improved by applying mirror therapy training and virtual therapy to lower extremities.

Several studies have demonstrated that mirror therapy and functional electrical stimulation have a positive effect on the recovery of stroke patient function. However, in mirror therapy, it is difficult to perform spontaneous muscle contraction on the paralyzed side of a stroke patient. In addition, it has been reported that functional electrical stimulation in the form of simple passive repetition can decrease the effect of motor relearning, which is important in rehabilitation of stroke patients [24]. Therefore, as an intervention method to compensate for this, mirror therapy in parallel with EMG-Triggered functional electrical stimulation has been devised. There is a lack of research on the balance and gait of stroke patients.

Therefore, this study investigates the effect on balance and gait ability of chronic stroke patients through an experimental study on mirror therapy with EMG-Triggered functional electrical stimulation, and the reference data for interventions are also provided to improve balance and gait ability of stroke patients.

## 2. Materials and Methods

### 2.1. Subjects

The study was conducted on those who met the selection criteria among chronic stroke patients who visited B Hospital and N Hospital in Gyeonggi Province and agreed to participate in this study. After completing the consent form, 60 subjects who met the selection criteria were selected. Participants in the study received sufficient explanations on the progress of the study and agreed in writing to the progress of this study. Selection criteria were as follows; (1) Patients who had been diagnosed with a stroke for the first time for 6 months or more, (2) patients without visual or hearing impairments, (3) patients without cognitive impairment with a Korean version of mini status examination (K-MMSE) score of 24 or higher, (4) patients who could walk independently, (5) patients and their guardians who understood the purpose of this study and agreed to participate in this study, (6) patients who satisfied all of the above. Exclusion criteria were as follows: (1) patients with unilateral neglect, (2) patients with somatosensory deficits that might affect the intervention, (3) patients with musculoskeletal damage and degenerative diseases that might affect balance and gait.

### 2.2. Protocol

A total of 60 people were selected according to the screening criteria and randomly divided into three groups using a computer program (Use of the program on the MINZKN.com) before the start of training to minimize selection bias. According to the experimental method, 20 patients were assigned into each of the following three groups: (1) mirror treatment and functional electrical stimulation group (MT-EF), (2) mirror treatment group (MT), and (3) conservative treatment group (CON). Each group was trained 60 min a day, 5 times a week for 8 weeks. The pre-test was performed the week before intervention, and the post-test was performed after 8 weeks of training. Prior to the start of this study, participants were fully informed of the purpose of the study, contents of the study, and confidentiality. This study was conducted after informing participants that they could withdraw from the study at any time if desired. The recruitment and procedure of study subjects were deliberated by the Institutional Bioethics Committee of Gimcheon University (No.GU-201911-HRa-20-03).

#### 2.2.1. MT-FE Group

In the mirror therapy training group, with EMG-Triggered functional electrical stimulation, conservative physical therapy was performed 30 min once a day, 5 times a week for 8 weeks. In addition, mirror therapy training was performed 30 min once a day, 5 times a week for 8 weeks in parallel with EMG-Triggered functional electrical stimulation.

**Figure 1:**
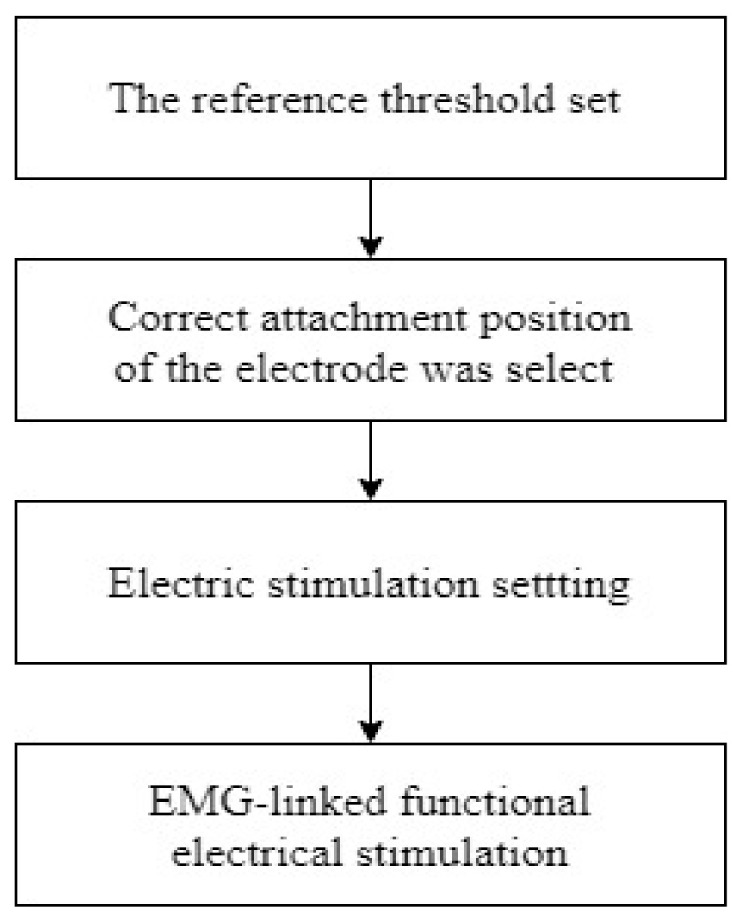
Diagram of EMG-FES stimulation.

Mirror therapy training was performed on a mat in an independent space. The patient was asked to sit properly in a chair with a backrest, taking a ready posture and receiving a mirror treatment program (visual feedback was only received as a mirror image of the non-paralyzed leg). The paralyzed leg was covered by a mirror to make it invisible. Mirror therapy using mirror nerve cells is a method using the visual illusion effect so that the paralyzed limb is moving normally through reflection in the mirror by moving the non-paralyzed limb of a stroke patient (Subjects freely perform flexion and extension exercises of the knee and ankle joint independently for 30 min in a chair sitting position). Simultaneously with a mirror therapy training, EMG-Triggered Functional Electrical Stimulation was applied to the paralyzed lower limb. In other words, the movement of the non-paralyzed lower limbs undergoing mirror treatment could provide a functional electric stimulation to the paralyzed leg through an EMG-Triggered functional electric stimulation device. Myomed 134 (Enraf-Nonius, Rotterdam, The Netherlands) was used as a training device for EMG-Triggered functional electrical stimulation. Myomed 134 is a device that learns self-regulation by viewing and feeling the body’s autonomic nervous system reactions such as muscle tone, brain waves, heart rate, and body temperature on a computer screen. EMG-Triggered electrical stimulation was performed with two surface electrodes (Paralyzed and non-paralyzed both). This electrode has two functions: Collecting EMG signals and transmitting electrical stimulation. The reference threshold was set according to the degree of voluntary muscle contraction of the paralyzed lower limb. The correct attachment position of the electrode was selected by electrically stimulating the surrounding synergic muscle (Quadriceps muscle, hamstring muscle, Tibialis anterior muscle, gastrocnemius muscle) until flexion and extension movements of the knee and ankle joints were observed. When electric stimulation occurred, it rose for 0.1 s, contracted for 5 s, and fell for 2 s. Stimulation with 10–20 mA, 35 Hz current was then performed. To minimize fatigue, a 5-s rest period was allowed between contractions. If the patient did not exceed the reference threshold, electric stimulation was automatically started after 20 s (Figure 1) [25].

#### 2.2.2. MT Group

In the mirror therapy training group, conservative physical therapy was performed 30 min once a day, 5 times a week for 8 weeks. Mirror therapy training was performed 30 min once a day, 5 times a week for 8 weeks. In the MT group, only mirror therapy was performed, excluding the EMG-Triggered electrical stimulation.

#### 2.2.3. Conservative Treatment Group (CON)

In the conservative treatment group, neurophysiological treatments such as neuro-developmental physical therapy (PNF, bobath approach) and conservative therapy (general active and passive joint movements and stretch movements, joint mobilization, muscle strength exercise, gait training) were applied to the paralyzed lower extremity for 30 min each time, twice a day, 5 times a week for 8 weeks.

### 2.3. Measurement

#### 2.3.1. Biorscue (COP, LOS)

To determine the subject’s static balance ability, the total travel distance of the center of pressure (COP) was measured while standing on a foot pressure sensor. As a measuring device, Biorecue (Analysis system by biofeedback, AP 1153 RM Ingenierie, Rodez, France) was used. Total COP movement distance was measured by allowing the subject to stand upright, spread his legs 30°, and look forward, and then stand with the center corrected for 1 min. In this evaluation, the smaller the COP travel distance, the smaller the body agitation, meaning that the balance ability was better. Three measurements were taken for all subjects and an average length was calculated. LOS (Limit of stability) measures the moving area by moving the center point of the human body in eight directions: Front, back, left, right, and diagonal using the one programmed in the measurement equipment. When the subject stood on the foot pressure sensor, an arrow in a specific direction appeared randomly on the computer screen. The subject then moved the center of the body in the direction of the arrow. At this time, both feet should always be placed on the foot pressure sensor. Measurements were taken from the beginning when the feet fell. COP was used as a measure of postural control. The measurement of the variable of the center of pressure represented the change in the point where the ground repulsion was synthesized, meaning the average weight of all pressure points in contact with the ground [26].

#### 2.3.2. Berg Balance Scale (BBS)

In this study, the Berg balance scale was used to measure the subject’s balance ability. The Berg balance scale consisted of a total of 14 items that could be divided into three areas: sitting, standing, and posture change. Each item had a minimum of 0 points and a maximum of 4 points. Thus, the highest possible score was 56 points. This measurement tool has high intra-measurers reliability (*r* = 0.99) and inter-measurers reliability (*r* = 0.97). Thus, it has high reliability and internal validity for evaluating balance ability [27,28]. The average point was recorded by measuring each subject three times.

#### 2.3.3. Functional Reach Test (FRT)

FRT measures the distance that can be reached horizontally by maintaining a stable support surface in a comfortable standing position and extending the arm forward. FRT is an economical, simple, and highly reliable test tool that can well measure the stability limit of a subject. This method was developed to detect balance disorders and examine changes in balance performance. Its inter-measurers reliability had *r* = 0.99 and its intra-measurers reliability had *r* = 0.98 [29].

#### 2.3.4. 10 m Walk Test (10 m WT)

For the 10 m walking test, a 14 m straight distance between two points was constructed as a walking path. The connection was made using a tape with a width of 10 cm. Each 2 m at the beginning and end was set as an acceleration and deceleration distance [30]. The walking time was measured with a stopwatch for a distance of 10 m. The measured time was calculated, together with the distance to obtain speed, and then the speed was used as a walking measurement variable. All subjects were measured three times and the average time was obtained as the result.

### 2.4. Statistical Methods

PASW Statistics 22.0 was used for all statistical analyses in this study. Normality was determined by the Shpiro-Wilk test. Among general characteristics of subjects, sex, stroke type, and paralyzed side were analyzed using Chi-square test. Age, height, weight, MMSE (Mini-mental state examination), and homogeneity of dependent variables before training among three groups were tested through one-way analysis of variance (ANOVA). The difference between before and after treatment in the group was measured through a paired *t*-test. To compare differences between groups, one-way ANOVA was performed. Bonferroni was used for intergroup post-test. All statistical significance levels of data were at *α* = 0.05 or less.

## 3. Results

Sixty subjects who participated in this study were randomly assigned to the MT-FE group, the MT group, and the CON group. There was no significant difference in the general characteristics of subjects among these three groups (*p* > 0.05) (Table 1). Table 2 shows results of change in balance and gait ability according to the intervention. All groups showed significant improvement within each group. For comparison between groups, the EF-MT group had significant more improvement than the MT group and the CON group (*p* < 0.05).

## 4. Discussion

The purpose of this study was to evaluate effects of interventions on balance and gait ability of chronic stroke patients. Three groups were used: (1) combined EMG-Triggered electrical stimulation and mirror therapy group (20 subjects); (2) mirror treatment group (20 subjects); and (3) conservative treatment group (20 subjects).

Balance problems are common after stroke onset and are associated with decreased mobility and increased risk for falls [30]. The most important training goals in stroke patient intervention, include obtaining independent activity and gait. For easier walking function, the balance ability of the standing posture and posture balance control should be given priorities [31]. In addition, in the early stages of motor learning of stroke patients, external feedback information is very important in rehabilitation of stroke patients [32].

Rizzolatti and Craighero [33] have mentioned that mirror-neuron meaning just seeing another person in action can make cells in that motion react as if they were acting. Mirror-neuron is known to play a very important role when learning new skills by understanding or observing other people’s behavior [34].

Balance ability in this study showed a significant increase in all three groups. In the comparison between groups, the MT-FE group showed a more significant improvement. There was no significant difference between the MT group and the CON group. This is consistent with results of previous studies. For example, Galeazzi et al. [20] reported that a stroke patient has improved balance ability by performing task training in front of a full-length mirror. Pinsault and Vuillerme [35] reported that the ability of weight transfer to the paralyzed side and postural control ability increase if a stroke-related hemiplegic patient is given a task training in front of a mirror. Robertson et al. [36] reported that BBS is significantly increased in the experimental group after performing balance and mobility training, with FES attached to stroke patients, compared to a control group, given balance and mobility training without FES attached. Electromyographic triggered electrical stimulation using EMG biofeedback is a treatment method that can improve a patient’s active will, unlike simple electrical stimulation that is performed passively. This is a treatment method that can induce self-regulation and active muscle contraction using biofeedback [37]. Movement observation contributes to the formation of the observed motor memory by promoting the reconstruction of the primary motor cortex, thus, promoting the formation of motor memory when combined with body movement [38]. Choi et al. [39] have reported that exercise image training using visual stimuli has an effect on weight transfer and dynamic balance, thereby improving symmetrical standing and stability. In the present study, the observation of motion through EMG-Triggered functional electrical stimulation and the mirror therapy were also considered to be causes of improvement.

Sütbeyaz et al. [13] have randomly allocated 40 stroke patients to a mirror treatment group and a phase treatment group who are then given mirror treatment in front of the mirror for the non-paralytic lower limb. As a result, the mirror treatment group showed significantly improved lower extremity motor function and walking ability. Kim et al. [40] have reported that after FES is applied in a sitting position to stroke patients, significant improvement is found in a waking up test. Yavuzer et al. [41] have reported that mirror therapy can aid the recovery of exercise and improve upper and lower extremity functions when it is applied to the upper and lower extremities of patients with subacute stroke. Bradley et al. [42] have reported that EMG biofeedback therapy is effective for active movement when it is added to physical therapy. Stevens and Stoykov [43] have reported that motion of the paralyzed side can be improved by transmitting visual information superimposed to the paralyzed side to the cerebrum of the non-paralytic side through mirror reflection in stroke patients. In the present study, gait ability was significantly increased in all three groups within each group after intervention. For comparison between groups, the MT-FE group showed a more significant improvement, consistent with results of the previous study. There was no significant difference between the MT group and the CON group. Movement observation can be said to be a process of understanding a certain type of behavior and learning movement by selectively imitating the part that is necessary for the patient [44]. Thirumala et al. [45] have suggested that observing spontaneous movement through a mirror can activate the bilateral inferior parietal area and the auxiliary motor area within the primary motor cortical area. They have reported that this activation can restore motor function through reorganization that replaces such function in other areas around the brain damaged by stroke. Biofeedback therapy has been used as an effective method to induce proper muscle contraction, maintain body alignment and normal movement by providing information about muscles or movements in real time [46]. Existing functional electric stimulation training is a method of inducing muscle contraction through passive stimulation with an open-chain control system. Unlike this, EMG-induced functional electric stimulation training proceeds with active intervention. In terms of the application method, many studies have reported that a functional electrical stimulation training with active movement is more effective in restoring upper limb function than a passive electrical stimulation therapy [47,48]. The results of the study also support this.

Since the present study was conducted on subjects who met the selection criteria, there was a limitation in generalizing and interpreting study results to balance and gait of all stroke patients. In addition, the effects of subject’s dependent variable cannot be excluded because the daily life of study subjects cannot be completely controlled. Studies on various variables related to gait are lacking. In the future, various studies need to be conducted that consider the diversification of mirror therapy training, conformity and adaptability of subjects to electrical stimulation, and various balance and gait evaluation tools. In particular, studies on gait parameters will be helpful in clarifying the therapeutic effects.

## 5. Conclusions

This study was conducted by combining EMG-Triggered functional electrical stimulation and mirror therapy, in order to investigate their effects on balance and gait ability of chronic stroke patients. The results showed a positive effect on balance and gait ability of chronic stroke patients. Restoration of balance and walking ability is very important for the daily life of chronic stroke patients. The results of this study suggest that brain plasticity stimulation can be more stable and easily given to stroke patients by combining EMG-Triggered functional electrical stimulation and mirror therapy. This is significant as an intervention method for stroke patients requiring long-term treatment.

## Figures and Tables

**Table 1:** The general characteristics of the subjects (*n* = 60).

Variables	MT-EF Group (*n* = 20)/M ± *SD*	MT Group (*n* = 20)/M ± *SD*	CON Group (*n* = 20)/M ± *SD*	F	*p*
Age (year)	56.05 ± 14.43	57.45 ± 5.27	59.70 ± 8.65	0.655	0.523
Height (cm)	163.91 ± 6.96	164.67 ± 8.69	161.09 ± 7.80	1.154	0.323
Weight (kg)	62.80 ± 7.59	64.52 ± 8.04	64.09 ± 9.71	0.222	0.802
MMSE (score)	27.05 ± 1.64	27.30 ± 1.45	27.70 ± 1.95	0.750	0.477
Gender (male/famale)	12/8	9/11	10/10	0.934	0.627
Diagnosis (I/H)	11/9	5/7	8/12	0.934	0.627
Affected side (Left/Right)	9/11	10/10	9/11	0.134	0.935
COP Length (cm)	31.65 ± 3.28	36.60 ± 6.82	33.15 ± 4.56	1.838	0.221
LOS (cm^2^)	5948.05 ± 2242.04	5691.00 ± 2075.61	5417.45 ± 2369.64	0.283	0.799
BBS (score)	39.50 ± 5.88	39.30 ± 6.12	38.40 ± 4.85	0.216	0.605
FRT (cm)	17.15 ± 7.73	16.35 ± 6.60	14.81 ± 7.68	0.523	0.605
10 m walk test (m/s)	0.62 ± 0.27	0.74 ± 0.34	0.66 ± 0.31	0.783	0.605

MT-EF, Mirror Therapy and EMG-FES; MT, Mirror Therapy; CON, Consevative therapy, MMSE, mini-Mental State Examination; I, infarction; H, hemorrhage; COP length, center of pressure length; LOS, Limited of stability; BBS, berg balance scale; FRT, functional reach test; General characteristics and dependent variables are calculated by one way ANOVA and Chi-squared test.

**Table 2:** Comparison pre and post test among three groups (*n* = 60).

Variables	MT-EF Group (A) (*n* = 20)/M ± *SD*	MT Group (B) (*n* = 20)/M ± *SD*	CON Group (C) (*n* = 20)/M ± *SD*	F	*p*
COP Length (cm)					
pre	31.65 ± 3.28	36.60 ± 6.82	33.15 ± 4.56	1.838	0.221
post	26.96 ± 3.29	33.54 ± 7.05	30.16 ± 4.05		
change	4.69 ± 0.76	3.06 ± 0.85	2.30 ± 2.21	10.009	0.000 A|BC
t	27.518163	16.087682	6.068127		
*p*	0.000	0.000	0.000		
LOS (cm^2^)					
pre	5948.05 ± 2242.04	5691.00 ± 2075.61	5417.45 ± 2369.64	0.283	0.799
post	7127.90 ± 2400.43	6218.75 ± 2082.27	5841.20 ± 2465.15		
change	−1179.85 ± 1036.26	−527.75 ± 204.97	−423.75 ± 237.93	8.601	0.001 A|BC
t	−5.091808	−11.514431	−7.964972		
*p*	0.000	0.000	0.000		
BBS (score)					
pre	39.50 ± 5.88	39.30 ± 6.12	38.40 ± 4.85	0.216	0.605
post	44.05 ± 5.87	42.45 ± 5.92	41.55 ± 4.83		
change	−4.55 ± 2.24	−3.15 ± 1.42	−3.15 ± 1.50	4.231	0.019 A|BC
t	−9.102396	−9.889860	−9.413531		
*p*	0.000	0.000	0.000		
FRT (cm)					
pre	17.15 ± 7.73	16.35 ± 6.60	14.81 ± 7.68	0.523	0.605
post	22.30 ± 8.07	18.15 ± 6.42	18.30 ± 7.78		
change	−5.15 ± 1.18	−1.80 ± 2.65	−3.49 ± 1.61	15.300	0.000 A|BC
t	−19.483491	−3.040270	−9.695429		
*p*	0.000	0.007	0.000		
10 m walk test (m/s)					
pre	0.62 ± 0.27	0.74 ± 0.34	0.66 ± 0.31	0.783	0.605
post	0.82 ± 0.3	0.84 ± 0.38	0.72 ± 0.30		
change	−0.19 ± 0.13	−0.10 ± 0.13	−0.07 ± 0.06	6.909	0.002 A|BC
t	−6.840639	−3.492978	−5.285515		
*p*	0.000	0.002	0.000		

MT-EF, Mirror Therapy and EMG-FES; MT, Mirror Therapy; CON, Consevative therapy, COP length = center of pressure length; LOS = Limited of stability; BBS = berg balance scale; FRT = functional reach test; A|BC = Group A is significantly different from Groups B and C.
